# The Dutch Mini-Linguistic State Examination (MLSE-NL): findings in Primary Progressive Aphasia (PPA)

**DOI:** 10.1007/s00415-026-14203-3

**Published:** 2026-10-23

**Authors:** Rosie Coppieters, Nikki Janssen, Peter Garrard, Fenna Hiemstra, Annelien Duits, Mattias De Coninck, Stéphanie De Keulenaer, Ian Storey, Vitória Piai, Johan Renssen, Carla Ruis, Harro Seelaar, Esther van den Berg, Sara Van Mossevelde, Rose Bruffaerts, Lize Jiskoot

**Affiliations:** 1 Experimental Neurobiology Unit, University of Antwerp, Antwerp, Belgium; 2 VIB Center for Molecular Neurology, University of Antwerp, Antwerp, Belgium; 3 Department of Psychiatry and Medical Psychology, Radboud University Medical Center, Nijmegen, The Netherlands; 4 Department of Neuroscience, City St George’s, University of London, London, UK; 5 ALS Center Netherlands, University Medical Center Utrecht, Utrecht, The Netherlands; 6 Department of Psychiatry and Psychology, Maastricht UMC+, Maastricht, The Netherlands; 7 Centrum voor Neuropsychologie, Antwerp, Belgium; 8 Donders Institute for Brain, Cognition and Behavior, Radboud University, Nijmegen, The Netherlands; 9 Department of Neurology, Alzheimer Center Amsterdam, Amsterdam UMC Location VUmc, Amsterdam, The Netherlands; 10 Department of Neurology and Neurosurgery, University Medical Center Utrecht, Utrecht, The Netherlands; 11 Department of Experimental Psychology, Utrecht University, Utrecht, The Netherlands; 12 Department of Neurology and Alzheimer Center, Erasmus MC University Medical Center, Rotterdam, The Netherlands; 13 Neurology Department, Antwerp University Hospital (UZA), Antwerp, Belgium

**Keywords:** Dementia, Diagnosis, Neuropsychology, Cross-linguistic, Aphasia, Semantic

## Abstract

**State of the art:**

Changes in speech and language occur as the first symptoms of Primary Progressive Aphasia (PPA). The Mini-Linguistic State Examination (MLSE) is a short speech and language screening test used in the diagnostic process of PPA in English-speaking patients. We adapted the MLSE into Dutch (MLSE–NL) and validated this new version as a diagnostic screening tool in Dutch-speaking patients.

**Methodology:**

We assessed the MLSE–NL in 90 Dutch-speaking patients with PPA (nonfluent variant PPA (nfvPPA) *n* = 23, logopenic variant PPA (lvPPA) *n* = 24, semantic variant PPA (svPPA) *n* = 32, PPA Not Otherwise Specified (PPA–NOS) *n* = 11) (mean age: 68.3, 47.8% male) and 276 healthy volunteers (mean age: 56.3, 38.8% male). The MLSE–NL includes subscores in motor speech, phonological structure, semantic knowledge, syntax, and working memory. Group differences were assessed using Kruskal–Wallis tests, with linear models controlling for covariates as confirmatory analyses.

**Results:**

The MLSE–NL shows significantly lower scores for all PPA variants for total score, ranging from 30 to 96/100 in patients and 83 to 100/100 in controls. The AUC for total score was 0.99 (subdomain AUCs ranged from 0.80 to 0.92). nfvPPA had more motor speech errors than controls, svPPA had more semantic errors, while lvPPA had the most working memory deficits. Syntax errors were frequent in all variants, while phonological deficits were greater in lvPPA and nfvPPA. PPA–NOS had deficits in all domains aside from motor speech.

**Conclusions:**

The MLSE–NL can successfully discriminate the three canonical variants of PPA and PPA–NOS from controls and captures domain-specific neurolinguistic profiles.

**Supplementary Information:**

The online version contains supplementary material available at https://doi.org/10.1007/s00415-026-14203-3.

## Introduction

Primary progressive aphasia (PPA) is a neurodegenerative syndrome characterized by a progressive deterioration of speech and/or language abilities. Current consensus criteria distinguish three canonical variants, nonfluent/agrammatic (nfvPPA), semantic (svPPA), and logopenic (lvPPA), based on domain-specific linguistic impairments. However, a substantial part of PPA cases cannot be classified into a specific subtype and is referred to as “Not Otherwise Specified” (PPA–NOS) [1]. Because the current diagnostic methods for PPA are often not readily available, are extensive, and can require specialist expertise, other screening tools are being investigated for diagnosis and differentiation. Neuropsychological tests that examine speech and language functioning are currently used clinically. However, speech and language testing often requires an in-depth knowledge of the tests and how to carry them out, as well as a knowledge of potential speech and language deficits and how to categorize them. For this reason, shorter and simpler tests are required for screening and referral purposes. Though most tools used for screening focus on a single domain such as confrontation naming or fluency, but do not assess the full width of language.

The PPA literature remains heavily weighted toward English-speaking cohorts [2, 3], and many language assessments have been developed and normed primarily in the English language. This imbalance creates challenges for clinicians and researchers working with patients that speak other languages, who often lack validated tools and robust normative datasets for accurate diagnosis and variant classification. Importantly, cross-linguistic differences are not merely logistical: there is emerging evidence that the clinical presentation of PPA can vary with the native language spoken [4–6]. The Mini-Linguistic State Examination (MLSE) is a novel speech and language test developed initially in English to diagnose both speech and language deficits in PPA. Currently, the MLSE is available in a limited number of languages and has been formally validated only in English, Spanish, and Greek [7–9]. The MLSE characterizes patients based on their scores in different speech and language domains, including motor speech, phonological structure, semantic knowledge, syntax, and phonological working memory [7]. The original English version of the MLSE shows high diagnostic accuracies for each of the three variants of PPA, with area under the curves of 0.92 for lvPPA, 0.93 for svPPA, and 0.98 for nfvPPA. The MLSE is also in the process of being validated in a number of other languages, and translations are already available in many more. As of yet, no results have been published on a translation of the MLSE in Dutch and it has not yet been used in Dutch-speaking patients.

In order to diagnose and inform prognosis in neurodegenerative diseases such as PPA, the development of tools such as the MLSE in languages other than English is vital. For (future) clinical trials this will also be particularly relevant to assess PPA patients outside of English-speaking countries. The aim of this study is to adapt the MLSE into Dutch (MLSE–NL) and validate the new version in Dutch-speaking patients with PPA (nfvPPA, lvPPA, svPPA, PPA–NOS) and a large group of healthy controls to provide a normative dataset for future usage in a clinical setting.

## Methods

### Participants

90 patients with PPA were recruited from academic outpatient memory clinics after a clinical visit, and were classified a priori into a PPA subtype (nfvPPA = 23, lvPPA = 24, svPPA = 32 according to the Gorno-Tempini et al. 2011 criteria or PPA–NOS = 11 based on the roadmap outlined by Marshall et. al. [10]) using all available clinical information (e.g., patient- and informant-based information, neuropsychological and language tests, structural MR brain imaging, fluid biomarkers). All patients had sporadic PPA or had not undergone genetic testing. Five of the patients with lvPPA had lumbar puncture data available, confirming Alzheimer’s Disease pathology in all 5 cases (at least A+T+ according to the ATN classification [11]. Two patients with lvPPA had concomitant Corticobasal Syndrome (CBS) (17 of whom data were available for), and seven patients with nfvPPA had concomitant CBS or Progressive Supranuclear Palsy (PSP) (19 of whom data were available for). Academic outpatient memory clinics included the Department of Neurology of Antwerp University Hospital (Antwerp, Belgium), the Department of Neurology and Alzheimer Center of the Erasmus MC University Medical Center (Rotterdam, the Netherlands), Alzheimer Centrum Amsterdam UMC (Amsterdam, the Netherlands), and Radboudumc (Nijmegen, the Netherlands). Participants were recruited only if they were able to provide written informed consent. The study adhered to all requirements for the provisions of the Declaration of Helsinki (World Medical Association, Edinburgh, 2000). Approval was issued by the Committee for Medical Ethics UZA–UA (BUN B3002022000114) and the Erasmus University Medical Center ethics committee (MEC-2023-0724). 276 healthy controls were recruited via social media advertisements, poster advertisements, local research cohorts, and word of mouth. Exclusion criteria for controls included a history of severe neuropsychiatric or developmental disorders including dyslexia that could interfere with language or cognitive performance. Controls were required to speak Dutch at the level of a native speaker. The Montreal Cognitive Assessment (MoCA) and Boston Naming Test (BNT) were also collected in patients and controls to assess cognitive function in controls and as a later comparison with the MLSE–NL.

### MLSE–NL

The MLSE–NL is composed of 11 subtests assessing five different domains of speech and language, namely, motor speech, phonological structure, semantic knowledge, syntax, and verbal working memory. The subtests are: (1) a naming test, (2) a repetition test with syllables and a multisyllabic word, (3) a repeat-and-point test, (4) repetition of non-words, (5) a semantic association test, (6) a verbal sentence-comprehension test, (7) a sentence-comprehension test in which the participant is required to match one of the four images to a spoken statement, (8) a reading test with real and non-words, (9) a sentence repetition test with multisyllabic sentences of increasing length, (10) a writing test, and (11) a picture description test. The subtests are scored for motor speech distortions, phonological errors, semantic errors, syntactic errors, working memory errors, and for non-responses. Motor speech and phonological structure are scored out of 30, semantic knowledge out of 20, and syntax and working memory out of 10, giving a total score out of 100, with higher scores indicating better performance. The numbers and distribution of errors give insight into the nature of the speech and language deficits. The MLSE can be administered in 15–20 min.

**Box 1:** Description of the different tasks of the MLSE

Subtask	Description	Domains Scored
1. Naming	Black-and-white drawings of typical and atypical objects	Semantic knowledge, motor speech, phonological structure
2. Repetition	Repetition of syllables and multisyllabic words	Motor speech, Phonological structure
3. Repeat-and-point	Repeat target word and identify matching image among distractors	Motor speech, Phonological structure [repetition], Semantic knowledge [pointing]
4. Non-word repetition	Repetition of pseudowords	Motor speech, Phonological structure
5. Semantic association	Match semantically related images	Semantic knowledge
6. Sentence comprehension I	Answer questions about spoken and written sentences	Syntax
7. Sentence comprehension II	Select image matching spoken and written sentences	Syntax
8. Reading	Reading of words and non-words	Motor speech, Phonological structure, Semantic knowledge
9. Sentence repetition	Repetition of increasingly complex sentences	Motor speech, Phonological structure, Working memory
10. Writing	Written description of tooth brushing within 2 min	Syntax
11. Picture description	Description of seaside scene for 1 min	Motor speech, Phonological structure, Semantic knowledge, Syntax

### Adaptation into Dutch

First, two separate MLSE–NL versions were created based on independent translations for the two variants of Dutch spoken in the Netherlands and Flanders (Belgium), which were later reconciled in one version and optimized after pilot testing [12]. During the adaptation into Dutch, we sought to keep the stimuli as similar to the English original version of the MLSE to allow comparison with the original version. The Dutch counterparts were chosen based on phonemes, syllable count, pronunciation difficulty, and word familiarity among the general Dutch population, following advice from an experienced researcher with experience in linguistics and language test development (VP). The selected Dutch items had the same frequency of use and age of acquisition as their English counterparts, as demonstrated in previous research [13, 14].

The pictorial stimulus content largely aligned with the English version in subtests, such as naming (task 1), semantic association (task 5), sentence comprehension II (task 7) and picture description (task 11). Line drawings were (re-)created using AI image generation with appropriate prompting. For the repeat-and-point test (ask 3) the target words were switched from “Dachshund” to “Dalmatian”, and “Chrysanthemum” to “Orchid”, as the word frequency of “Dachshund” and “Chrysanthemum” in Dutch is lower than English, and Dachshund is a loanword from German.

In tests that require speech production of single (non-)words, repetition (task 2), repeat-and-point (task 3) and non-word repetition (task 4), stimuli were matched to the original test with regard to number of syllables and strong–weak vs. weak–strong patterns of lexical stress which have been identified as a sensitive marker of apraxia of speech in Dutch [15] as well as English. For the word reading test (task 8), loanwords whose pronunciation could not be deduced clearly by orthography (irregular orthography-to-phonology mapping) were selected. Words were selected that are pronounced identically across regional variants of Dutch spoken in the Netherlands and Belgium. The repetition of non-words test (task 4) and reading of non-words test (task 8) required the generation of novel pseudowords as the English words could not be used. To generate pseudowords with the same subsyllabic structure and complexity as the English version, “Wuggy”, a multilingual pseudoword generator was used [16]. This allowed the production of the novel pseudowords still following the phonotactic constraints of Dutch.

For the sentence-comprehension test I and II (tasks 6 and 7) the names were altered to equally common Dutch equivalents. In the adapted sentence stimuli, the number of subordinate clauses and passive constructions was retained to preserve syntactic complexity. In the sentence repetition task (task 9), stimuli were adopted from English, leading to a slight increase in phonological working memory load in the Dutch version, with an average of one additional syllable per stimulus compared with the English version. During the study, the MLSE–NL was administered by researchers that received hands-on training and supervision by the team that developed the MLSE–NL.

### Statistical analysis

Group demographics are shown in Table 1. Age, sex, and years of education were compared across the whole PPA patient group and control group using Kruskall–Wallis tests and post hoc Dunn tests.

**Table 1:** Demographics of included controls and patients with PPA

	Healthy Controls	All PPA	nfvPPA	lvPPA	svPPA	PPA–NOS
*N*	276	90	23	24	32	11
Sex (% male)	38.8%	47.8%	21.7%	70.6%	53.1%	45.5%
Age (*M* ± *SD*)	56.3 ± 16.8	68.6 ± 7.4	71 ± 8.2	69.7 ± 6.8	65.7 ± 6.9	69.8 ± 6.4
Education years (*M* ± *SD*)	15.4 ± 2.7	15.3 ± 3.1	14 ± 3	16.2 ± 2.9	15.7 ± 3.1	14.8 ± 3.2
MoCA score/30 (*M* ± *SD*) (n available)	27.5 ± 2 (*n* = 276)	18.6 ± 5.2 (*n* = 55)	22.4 ± 2.8 (*n* = 17)	15.2 ± 4.5 (*n* = 16)	19 ± 3.6 (*n* = 17)	16 ± 8.6 (*n* = 6)
BNT/60 (*M* ± *SD*) (n available)	55 ± 3.3 (*n* = 256)	34.6 ± 15.7 (*n* = 50)	50.8 ± 5.6 (*n* = 13)	35.6 ± 14.0 (*n* = 15)	20.9 ± 9.2 (*n* = 17)	36.4 ± 15.4 (*n* = 5)
CDR plus NACC FTLD global score (*M* ± *SD*) (n available)	/	1.18 ± 0.50 (*n* = 54)	1.20 ± 0.57 (*n* = 20)	1.27 ± 0.53 (*n* = 13)	1.10 ± 0.39 (*n* = 15)	1.08 ± 0.49 (*n* = 6)
Disease duration in months (*M* ± *SD*) (n available)	/	37.4 ± 20.0 (*n* = 58)	37.2 ± 21.0 (*n* = 20)	40.2 ± 19.9 (*n* = 15)	35.1 ± 21.3 (*n* = 16)	36.9 ± 17.3 (*n* = 7)

*svPPA* semantic variant Primary Progressive Aphasia, *nfvPPA* nonfluent variant Primary Progressive Aphasia, *lvPPA* logopenic variant Primary Progressive Aphasia, *PPA–NOS* Primary Progressive Aphasia not otherwise specified, *MoCA* Montreal Cognitive Assessment, *BNT* Boston Naming Test, *CDR plus NACC FTLD* Clinical Dementia Rating plus National Alzheimer’s Coordinating Center Frontotemporal Lobar Degeneration rating [17]. Disease duration: months since symptom onset

MLSE–NL total score and domain scores (motor speech, phonological structure, semantics, syntax, and phonological working memory) were compared between groups using linear models accounting for the covariates age, sex, and years of education, and by comparing the estimated marginal means with Bonferroni correction. We determined inter-rater reliability in a large subset of participants (*n* = 123; controls *n* = 64, PPA *n* = 59). Receiver Operator Curves (ROC) were calculated for each PPA variant for each MLSE–NL domain as well as the total score, to determine sensitivity and specificity as well as overall discriminative accuracy for each PPA variant from controls. To assess whether diagnostic performance varied according to disease stage, we performed a sensitivity analysis by stratifying PPA patients across variants using the global score of the CDR plus NACC FTLD [17] (Table 1; available in 54 patients) and calculating overall discriminative accuracy per disease stage (CDR plus NACC FTLD = 0.5 (prodromal), *n* = 7; CDR plus NACC FTLD = 1 (mild), *n* = 34; CDR plus NACC FTLD = 2 (moderate), *n* = 13).

### Machine-learning classification

A Random Forest (RF) classifier was implemented in Python (version 3.13.3) using the scikit-learn library (version 1.6.1) to classify diagnostic groups based on MLSE–NL domain scores. RF was selected due to its strong performance in medical classification problems, robustness to noise, ability to model nonlinear decision boundaries, and relative resistance to overfitting. The Python script used for this classification was taken from the English publication of the MLSE in order to allow for a direct comparison to the English findings [7].

The control group was reduced to minimize the class imbalance, since it was much larger than the patient group compared to the English version. 25 age- and education-matched controls were selected, with an even sex split. These 25 controls were selected based on having the closest Euclidean distance to the mean demographics of the patient groups and, therefore, considered the best match with the patient group in terms of age, sex, and years of education (see Supplementary materials for demographics of the 25 selected controls). The RF classifier was run including only the three canonical variants of PPA and then again including the PPA–NOS group as an exploratory analysis. All model development procedures were conducted exclusively on the training data. Within the training set, stratified k-fold cross-validation (k = 3) was performed to estimate internal model performance. Balanced accuracy and macro-averaged F1 score were computed for each fold, and mean ± standard deviation across folds were reported.

The RF classifier was constructed using the five MLSE–NL domain scores as predictors. Missing numeric values were imputed using median imputation within a preprocessing pipeline to prevent data leakage. No feature scaling was applied, as tree-based methods are invariant to monotonic transformations of predictor variables.

The RF model consisted of 100 decision trees. At each split, the number of predictors considered was set to the square root of the total number of features (scikit-learn’s default for classification tasks), consistent with standard RF practice. To address potential class imbalance, class weights were automatically adjusted using balanced subsampling within each bootstrap sample. All other hyperparameters were left at their default settings (criterion = “gini”, max depth = None, bootstrap = True, max samples = None).

After cross-validation, the model was retrained on the full training set and subsequently evaluated on the independent holdout dataset. Performance metrics included overall accuracy, balanced accuracy, classwise precision, recall, F1 score, and confusion matrices. In addition, one-versus-rest ROC curves were computed for each diagnostic group, and micro-averaged area under the curve (AUC) was calculated to summarize multiclass discrimination performance.

To interpret model predictions, SHAP (SHapley Additive exPlanations) values were computed for all test-set cases using a TreeExplainer, yielding per-class, per-feature attributions for each prediction. Global feature importance was summarized as the mean absolute SHAP value per feature within each diagnostic class and visualized as a grouped bar chart. To visualize the model's decision space, a two-component principal component analysis (PCA) was fitted on the preprocessed training-set features. Training cases were plotted as a background reference (colored by true diagnosis), and test-set cases were overlaid, distinguishing correctly classified cases from misclassified cases (annotated with their true and predicted labels), to characterize, where classification errors occurred relative to the overall diagnostic group structure.

## Results

### Demographic and clinical data

Demographic and clinical data are displayed in Table 1. Age differed significantly across groups (Kruskal–Wallis H (4) = 49.20, *p* < 0.001, η^2^H = 0.13), and was significantly lower in controls than all patient groups (all Dunn post hoc *p* < 0.001, *r* = 0.34–0.56). This was due to the fact that there was no age restriction in recruitment of controls, with the goal of developing a normative dataset across a wide range of age groups (18–85). No significant age differences were found between patient groups. The proportion of male and female participants differed significantly across groups (χ^2^(4) = 11.7, *p* = 0.019, Cramér's V = 0.18; Fisher’s exact *p* = 0.018), with more women among patients with nfvPPA and more men among patients with lvPPA and svPPA relative to controls. Years of education did not differ significantly across groups (H(4) = 6.63, *p* = 0.157, η^2^H = 0.007). MoCA scores differed significantly across groups (H(4) = 121.24, *p* < 0.001, η^2^H = 0.32) and were significantly lower in all patient groups than controls (all Dunn *p* < 0.001). There were no significant pairwise differences in MoCA scores between patient groups. BNT scores also differed significantly across groups (H(4) = 92.50, *p* < 0.001, η^2^H = 0.25 [large]) and were significantly lower in all patient groups than controls (all Dunn *p* ≤ 0.004). Among patient groups, BNT scores were significantly lower in patients with svPPA than in patients with nfvPPA (Dunn *p* = 0.040, *r* = 0.39), consistent with the prominent anomia characteristic of svPPA. No significant differences in CDR plus NACC FTLD global score or disease duration were found between patient groups.

### Normative data healthy controls

**Fig. 1:**
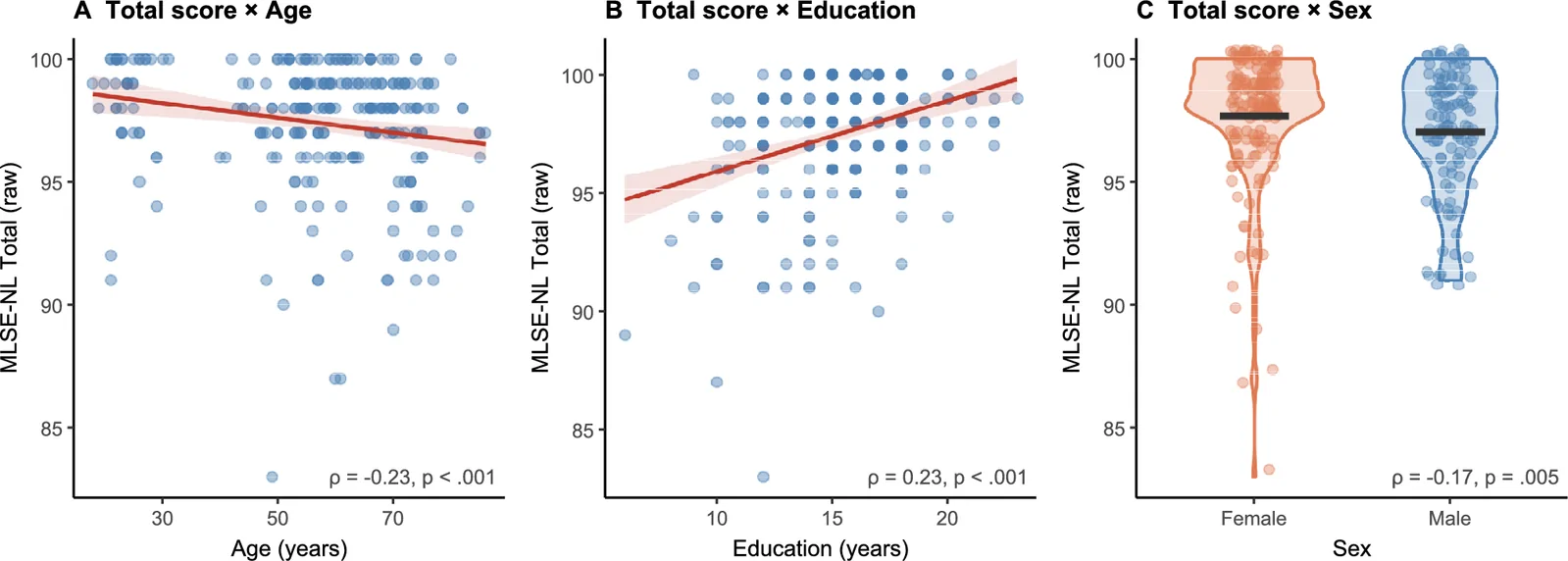
MLSE–NL total score by demographic variables in healthy controls. Linear regression line ± 95% CI

**Fig. 2:**
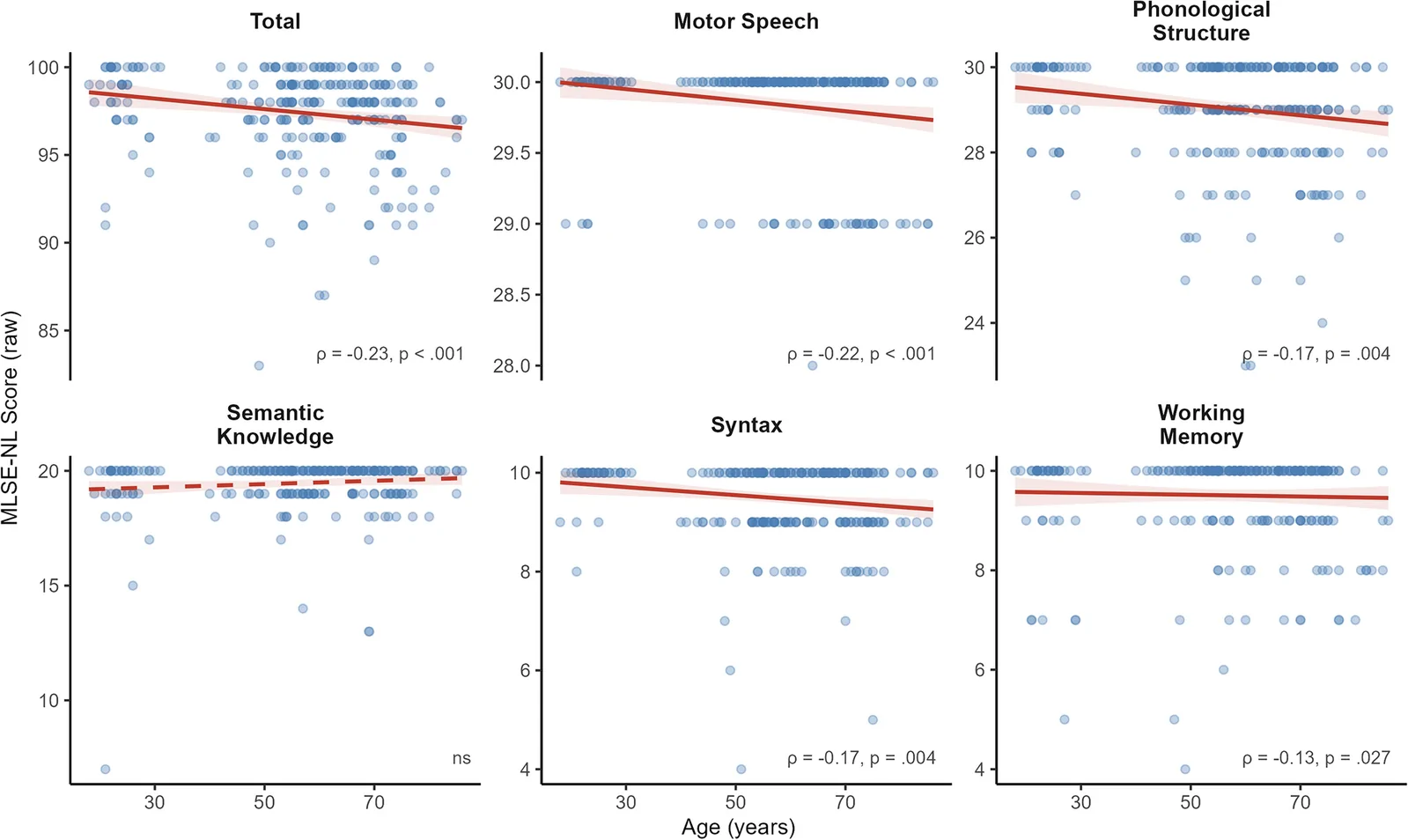
MLSE domains scores x age in healthy controls. Linear regression line ± 95% CI. Dotted line indicates non-significance

**Fig. 3:**
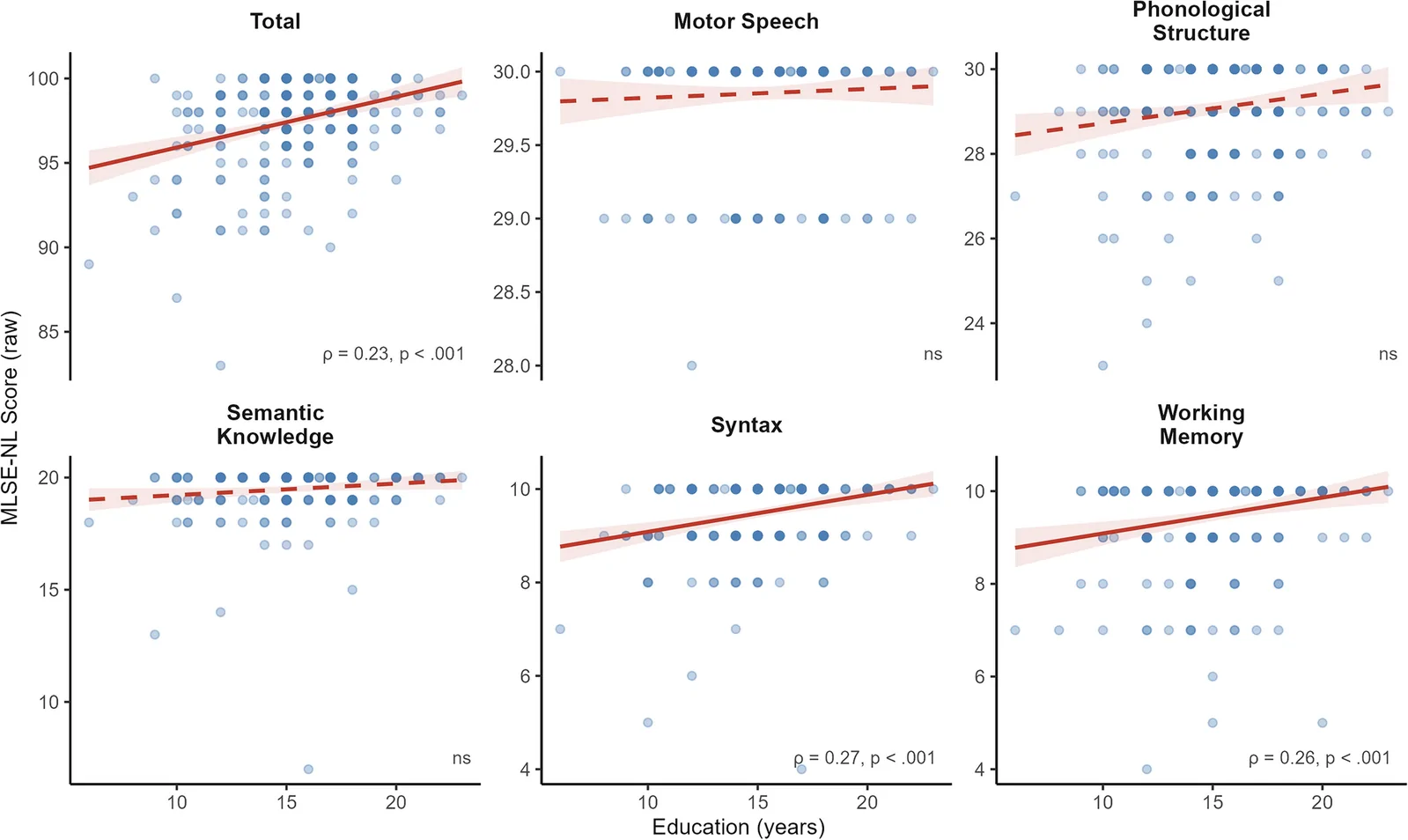
MLSE domains scores x education in healthy controls. Linear regression line ± 95% CI. Dotted line indicates non-significance

Spearman correlations indicated that total MLSE–NL score was significantly associated with age (*ρ* = −0.23, *p* <0.001), sex (*ρ* = −0.17, *p* = 0.005), and years of education (*ρ* = 0.23, *p *< 0.001), with older age and male sex associated with lower scores, and more years of education associated with higher scores. The clear effects of particularly age (Table 2) and years of education (Table 3) on MLSE–NL total scores suggest that age and education adjustment may improve the interpretation. At the domain level, motor speech was negatively correlated with age (*ρ* = −0.22, *p* < 0.001), but not with sex or years of education. Phonological structure was associated with age (*ρ* = −0.17, *p* = 0.004) and sex (*ρ* = −0.17, *p* = 0.004), with males having lower scores than females. Semantic knowledge showed a small but significant association with sex only (*ρ* = −0.14, *p* = 0.023), with males having lower scores than females. Syntax and working memory were each associated with age (*ρ* = −0.17, *p* = 0.004; *ρ* = −0.13, *p* = 0.027, respectively) and years of education (*ρ* = 0.27, *p* < 0.001; *ρ* = 0.26, *p* < 0.001, respectively), but not with sex. Figure 1 shows the MLSE–NL total score plotted against age, education, and sex. Figures 2 and 3 show the MLSE–NL total and domain scores plotted against age and education, respectively. The Supplementary materials show the percentile scores and performance of the control group across age, sex, and years of education for the different MLSE–NL domain scores and the MLSE–NL total score.

**Table 2:** MLSE–NL total score by age group (controls)

Statistic	≤ 39 (* n* = 44)	40–49 (* n* = 21)	50–59 (* n* = 81)	60–69 (* n* = 63)	70–79 (* n* = 57)	80+ (* n* = 10)
Mean (SD)	98.3 (2.2)	96.6 (3.7)	97.8 (2.1)	97.7 (2.7)	96.4 (2.8)	96.2 (2.5)
Range	91–100	83–100	90–100	87–100	89–100	92–100
2-SD cutoff	94.0	89.2	93.5	92.3	90.9	91.2
5th pct	94.1	91.0	94.0	92.2	91.0	92.4

Values: mean (SD), range, 2-SD cutoff, 5th percentile

**Table 3:** MLSE–NL total score by education level (controls)

Statistic	≤ 11 years (*n* = 22)	12 years (*n* = 21)	13–15 years (*n* = 105)	16–17 years (*n* = 60)	> 17 years (*n* = 59)
Mean (SD)	95.3 (3.5)	96.1 (3.8)	97.7 (2.3)	98.2 (2.0)	97.9 (1.7)
Range	87–100	83–100	91–100	90–100	92–100
2-SD cutoff	88.3	88.5	93.1	94.1	94.5
5th pct	89.1	91.0	92.1	95.0	94.9

Values: mean (SD), range, 2-SD cutoff, 5th percentile

Spearman correlations were conducted to examine the relationship between MLSE–NL domain scores and MoCA and BNT within the normative control group. The MLSE–NL total score was significantly correlated with both MoCA (*ρ* = 0.27, *p* < 0.001) and BNT (*ρ* = 0.19, *p* = 0.010) scores, indicating that higher MLSE–NL performance was associated with better general cognitive functioning and confrontation naming ability. At the domain level, phonological structure (*ρ* = 0.21, *p* < 0.001), syntax (*ρ* = 0.19, *p* = 0.002), and working memory (*ρ* = 0.23, *p* < 0.001) were each significantly positively correlated with the MoCA score, while semantic knowledge showed a smaller but significant positive association (*ρ* = 0.15, *p* = 0.011). Motor speech was not significantly associated with the MoCA score (*ρ* = 0.095, *p* = 0.116). For BNT, semantic knowledge showed the strongest and only substantial correlation (*ρ* = 0.36, *p* < 0.001), which is consistent with the shared lexical-semantic demands of both tasks. Working memory also showed a small significant positive association with the BNT score (*ρ* = 0.18, *p* = 0.017), while phonological structure, motor speech, and syntax were not significantly correlated with BNT performance (all *p* > 0.05).

### MLSE–NL total and domain scores in patients with PPA

**Fig. 4:**
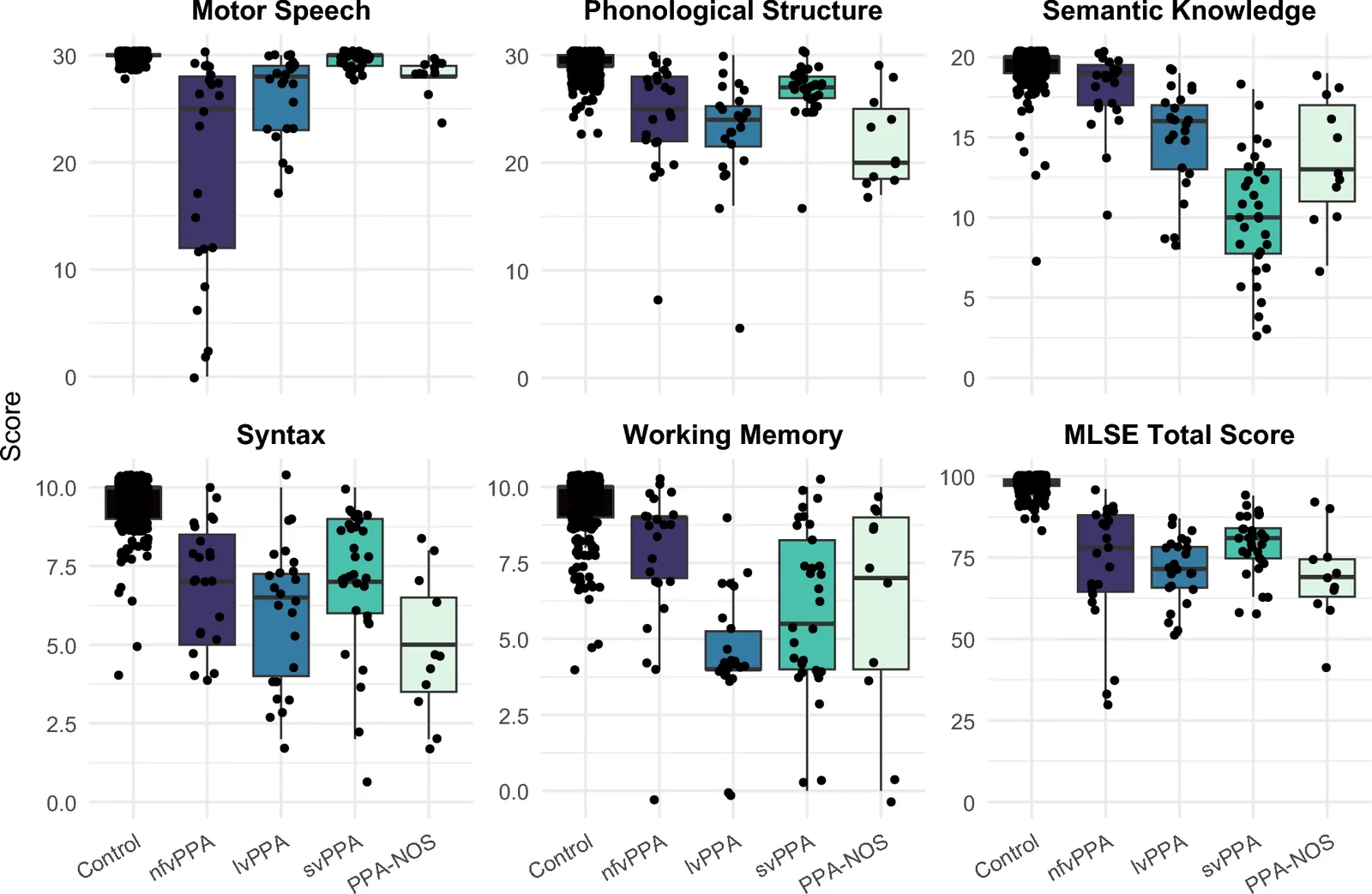
Domain scores and total scores per group. *svPPA* semantic variant Primary Progressive Aphasia, *nfvPPA* nonfluent variant Primary Progressive Aphasia, *lvPPA* logopenic variant Primary Progressive Aphasia, *PPA–NOS* Primary Progressive Aphasia not otherwise specified

**Fig. 5:**
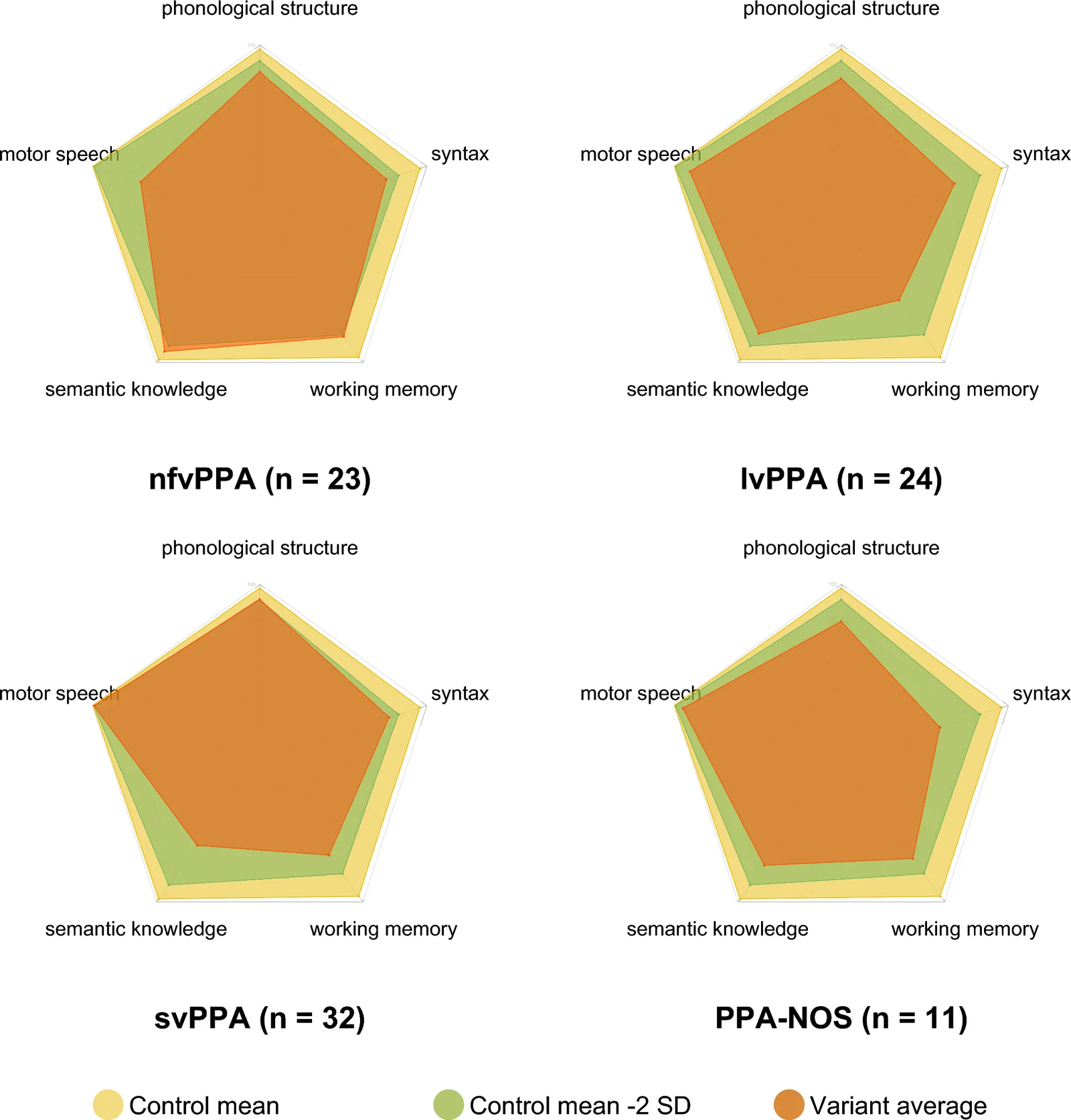
Radar plots showing the average domain scores of each patient group, displayed with mean control scores. *svPPA* semantic variant Primary Progressive Aphasia, *nfvPPA* nonfluent variant Primary Progressive Aphasia, *lvPPA* logopenic variant Primary Progressive Aphasia, *PPA–NOS* Primary Progressive Aphasia not otherwise specified

Based on linear models accounting for covariates (age, education, and sex), all patients had significantly lower MLSE–NL total scores than controls (*p* < 0.001), with no significant differences between PPA groups, see Fig. 4 for boxplot of domain and total scores for patients and controls and Fig. 5 for radar plots of domain scores in patient groups alongside mean control values.

Patients with nfvPPA showed widespread impairment compared to controls across all MLSE–NL domains (all *p* ≤ 0.0042), with a profile characterized by marked motor speech deficits, scoring significantly lower than patients with lvPPA and svPPA (both *p* < 0.0001). In contrast, semantic knowledge and working memory performance were relatively more preserved than in other PPA subtypes, with higher scores than patients with svPPA on the semantic knowledge domain (*p* < 0.0001) and higher scores than patients with lvPPA in the working memory domain (*p* < 0.0001).

Patients with lvPPA showed impairments across all MLSE–NL domains compared to controls (all *p* < 0.0001), with a profile characterized by reduced working memory and phonological structure. Working memory was significantly lower than in patients with nfvPPA (*p* < 0.0001) and svPPA (*p* = 0.0082), and phonological structure scores were also lower than in patients with svPPA (*p* < 0.0001).

Patients with svPPA demonstrated a distinct and selective semantic impairment, with significantly lower semantic knowledge scores than all other groups (all *p* < 0.0001). They also showed reduced performance in phonology, syntax, working memory, and the total score compared to controls (all *p* < 0.0001), but relatively preserved motor speech, scoring higher than in patients with nfvPPA (*p* < 0.0001).

Finally, patients with PPA–NOS exhibited widespread impairments compared to controls across all MLSE–NL domains except motor speech (all *p* < 0.0001; motor speech: *p* = 0.2269). Their performance across domains was generally intermediate, with higher semantic knowledge scores than patients with svPPA (*p* < 0.0001) and higher total scores than patients with svPPA (*p* = 0.0003) while not differing significantly from patients with lvPPA across MLSE–NL domains (all *p* ≥ 0.4030).

The radar plots in Fig. 5 illustrate these differences in variants by overlaying the average for each variant over the control mean and the control mean lowered by 2 standard deviations (calculated cutoff). In this way a comparison of the characteristic profiles of the variants with each compared to controls is shown.

Based on Koo & Li [18], the intraclass correlation coefficient (ICC) was good to excellent across MLSE–NL domain scores and the total score (Table 4). In the total sample, ICCs ranged from 0.78 to 0.97, with the highest reliability observed for the total score, motor speech, and semantic knowledge. Reliability was also excellent for syntax, and good for verbal working memory and phonological structure.

**Table 4:** Inter-rater reliability for the MLSE–NL: intraclass correlation coefficient (ICC) and 95% confidence interval (CI) for total and domain scores

Domain	*n*	ICC	95% CI
Total score	123	0.97	0.94–0.99
Motor speech	118	0.96	0.93–0.99
Phonology	118	0.78	0.57–0.97
Semantics	118	0.94	0.87–0.99
Syntax	119	0.91	0.74–0.99
Working memory	119	0.83	0.66–0.97

### Diagnostic and variant classification accuracy

#### ROC analysis

**Fig. 6:**
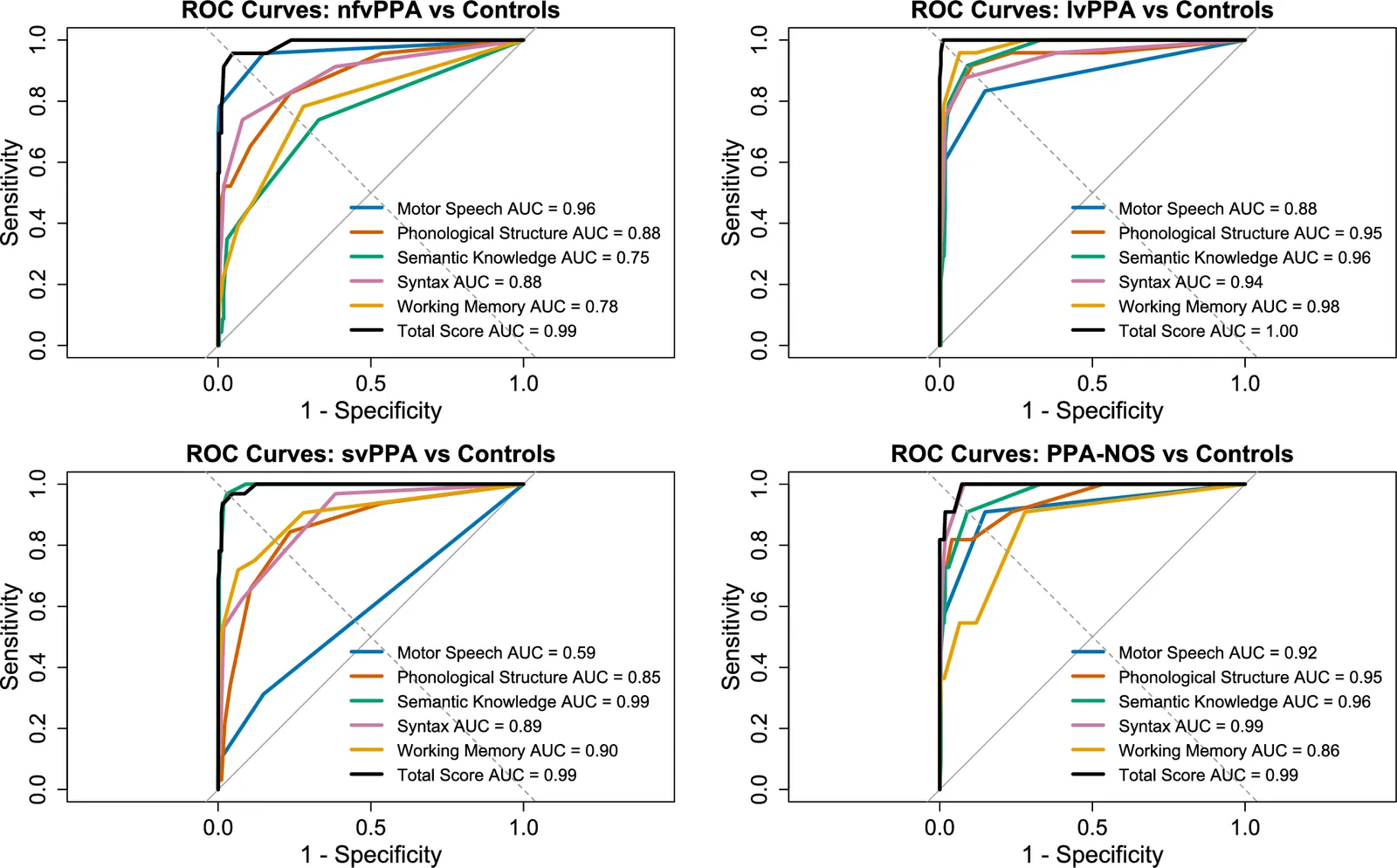
ROC curves for the domain scores for each patient group. *svPPA* semantic variant Primary Progressive Aphasia, *nfvPPA* nonfluent variant Primary Progressive Aphasia, *lvPPA* logopenic variant Primary Progressive Aphasia, *PPA–NOS* Primary Progressive Aphasia not otherwise specified

The results of the ROC analysis are shown in Fig. 6. The MLSE–NL total score was an excellent classifier for all patient groups vs. controls (AUC > 0.99 for all comparisons; svPPA: AUC = 0.99, 95% CI [0.99–1.00]; nfvPPA: AUC = 0.99, 95% CI [0.97–1.00]; lvPPA: AUC = 0.999, 95% CI [0.998–1.000]; PPA–NOS: AUC = 0.99, 95% CI [0.98–1.00]) (AUC > 0.99). In patients with nfvPPA, motor speech was the best at differentiating patients from controls (AUC = 0.96, 95% CI [0.91–1.00]). For patients with svPPA, the best differentiator was semantic knowledge (AUC = 0.99, 95% CI [0.99–1.00]). For patients with lvPPA, a number of MLSE–NL domains successfully differentiated from controls, including phonological structure (AUC = 0.95, 95% CI [0.88–1.00]), semantic knowledge (AUC = 0.96, 95% CI [0.94–0.99]), syntax (AUC = 0.94, 95% CI [0.88–1.00]) and working memory (AUC = 0.98, 95% CI [0.97–1.00]). For patients with PPA–NOS, motor speech (AUC = 0.92, 95% CI [0.82–1.00]), phonological structure (AUC = 0.94, 95% CI [0.87–1.00]), semantic knowledge (AUC = 0.97, 95% CI [0.93–1.00]), and syntax (AUC = 0.99, 95% CI [0.98–1.00]), successfully differentiated from controls.

Across all PPA variants, sensitivity analysis showed excellent discriminative accuracy vs. controls at every disease stage using the MLSE–NL total score: CDR plus NACC FTLD global score = 0.5 (AUC = 0.958, 95% CI [0.904–1.000]), CDR plus NACC FTLD global score = 1 (AUC = 0.998, 95% CI [0.995–1.000]), and CDR plus NACC FTLD global score = 2 (AUC = 0.999, 95% CI [0.997–1.000]).

### Random forest classifier

The predictive capacity of the RF model was good, with an overall accuracy of 0.74 and a balanced accuracy of 0.75 on the held-out test set. As shown in Table 5, a total of 11 out of 42 cases were misclassified.

**Table 5:** Confusion matrix for prediction using RF classification of PPA variants and controls carried out in 42 individuals

True\predicted	Control	lvPPA	nfvPPA	svPPA	Total
Control	9 (90%)	0 (0%)	1 (10%)	0 (0%)	10
lvPPA	0 (0%)	8 (80%)	1 (10%)	1 (10%)	10
nfvPPA	0 (0%)	3 (33%)	6 (67%)	0 (0%)	9
svPPA	1 (7.7%)	4 (30.8%)	0 (0%)	8 (61.5%)	13

Once PPA–NOS was included in the classifier the accuracy dropped to an overall accuracy of 0.652. PPA–NOS was misclassified in 3/11 cases, once as each variant nfvPPA, lvPPA, and svPPA. Two lvPPA, one nfvPPA, and one svPPA patient were misclassified as PPA–NOS

**Fig. 7:**
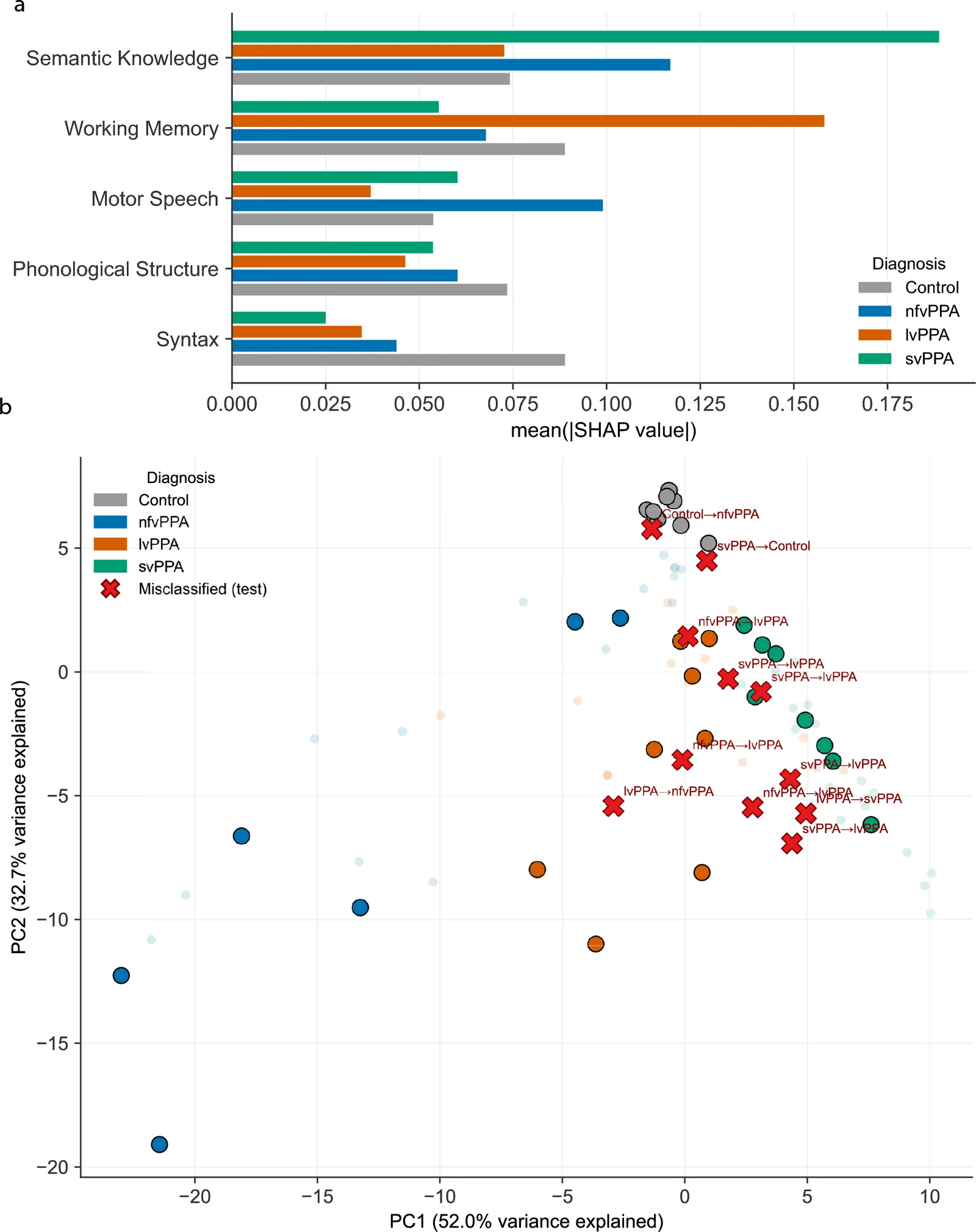
**a** Global SHAP feature importance by diagnostic class. **b** Principal component analysis (PCA) of MLSE score feature space to illustrate misclassifications

Diagnostic performance based on the RF model varied across syndromes. Controls were classified with a recall of 0.90 (9/10 correctly classified), with one case being misclassified as nfvPPA. SHAP analysis of this case indicated that despite ceiling-level semantic scores (20/20), consistent with a healthy control profile, slightly lower phonological structure scores (28/30) and working memory scores (9/10) relative to the control mean actively reduced the model's posterior probability for control below the base rate, drawing the classification toward nfvPPA, which had the most preserved semantic and working memory profile among the PPA subtypes. lvPPA was classified with a recall of 0.80 (8/10 correctly classified), with misclassifications of nfvPPA (*n* = 1) and svPPA (*n* = 1). nfvPPA achieved a recall of 0.67 (6/9 correctly classified), with all three errors involving misclassification as lvPPA. Inspection of these cases revealed anomalously high motor speech scores (27–29/30) relative to the nfvPPA mean (17.3/30), suggesting that very early motor speech abnormalities may not be captured by MLSE, leading to feature overlap with lvPPA. svPPA showed the lowest recall at 0.62 (8/13 correctly classified), with four cases misclassified as lvPPA and one as a control. The four svPPA cases misclassified as lvPPA shared reduced syntax (5.75/10 vs. 8.56/10) and working memory scores (3.75/10 vs. 7.00/10) relative to the remaining svPPA cases, placing them closer to the lvPPA feature profile, which is characterized by comparatively low syntax and working memory scores, see Fig. 7 for illustration of global SHAP feature importance and Random Forest misclassifications, with a Principal Component Analysis (PCA) of MLSE score feature space. PCs 1 and 2 explained 84.7% of the variance (with 100% being explained by 5 PCs). Motor speech and semantic knowledge contributed most to PCs 1 and 2 (0.813 and 0.711, respectively).

### Interpretable decision tree

**Fig. 8:**
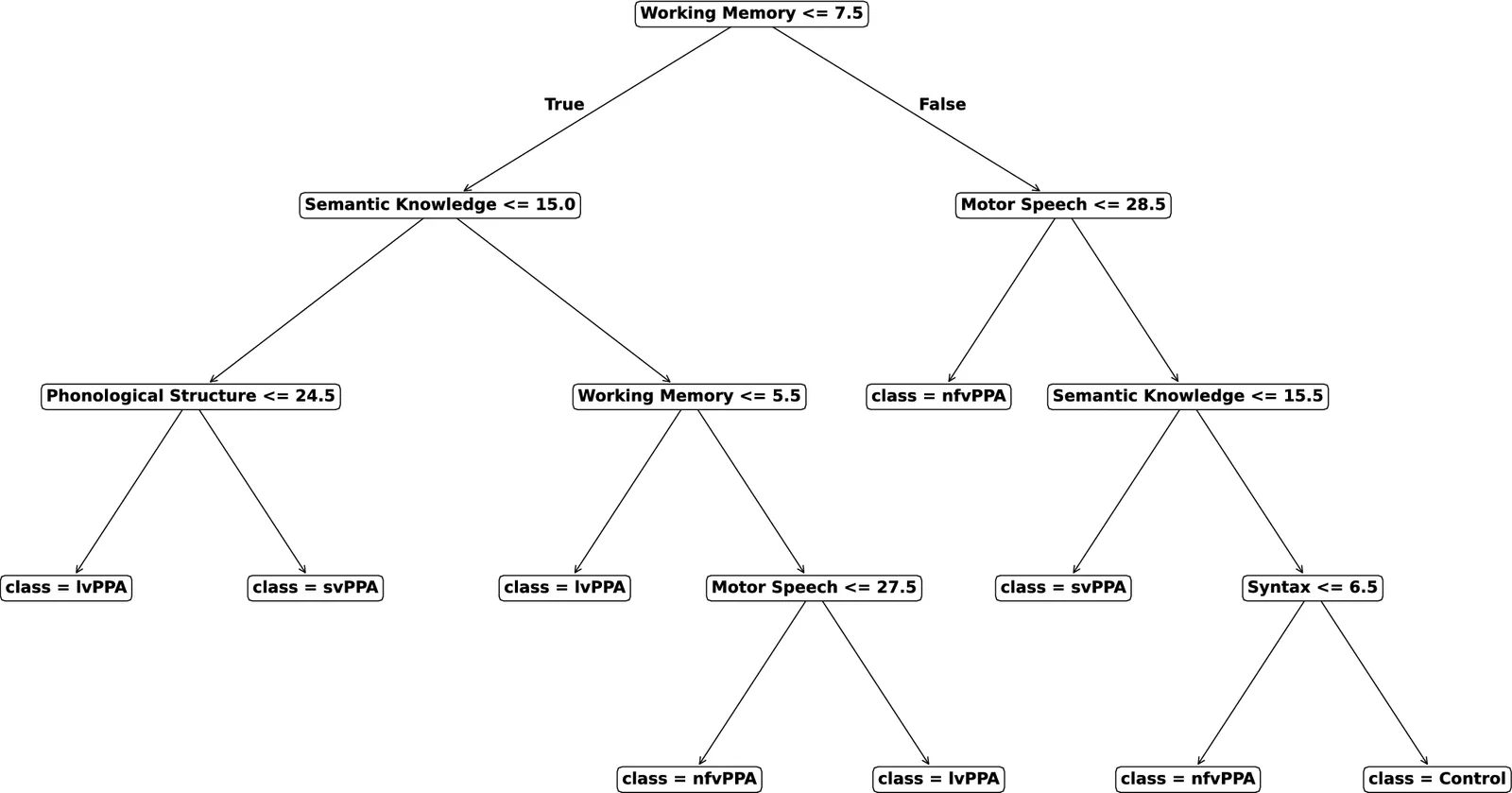
Data-driven decision tree selected from the random forest based on balanced accuracy and tree depth. *svPPA* semantic variant Primary Progressive Aphasia, *nfvPPA* nonfluent variant Primary Progressive Aphasia, *lvPPA* logopenic variant Primary Progressive Aphasia

In line with the approach of the original English MLSE, a representative decision tree (Fig. 8) was extracted post hoc from the RF, constrained by balanced accuracy and tree depth to maximize interpretability, as well as to determine possible differences in classification in the Dutch patient group. The tree performance on test set had a balanced accuracy of 0.83, with 10/10 controls correctly identified (100%), 8/10 lvPPA correctly identified (80%), 6/9 nfvPPA correctly identified (67%), and 11/13 svPPA correctly identified (85%). With the addition of the PPA–NOS group the balanced accuracy dropped to 0.66, in addition to the tree becoming more complex (see Supplementary materials for decision tree with PPA–NOS included).

## Discussion

In this study we adapted and validated the Dutch version of the MLSE (MLSE–NL). The MLSE–NL shows excellent diagnostic accuracy for discrimination between PPA patients and healthy controls.

The pattern of domain-specific impairments observed in the MLSE–NL closely reflects the expected cognitive-linguistic profiles of the canonical PPA variants, supporting the validity of the Dutch adaptation of the MLSE. Patients with svPPA demonstrated prominent semantic deficits, while lvPPA showed marked impairment in working memory (scoring lower in the sentence repetition task) and phonology, consistent with the underlying mechanisms proposed for these syndromes. Individuals with nfvPPA showed the expected predominant motor speech impairment together with syntactic difficulties, whereas patients with PPA–NOS exhibited a broader pattern of deficits spanning all domains aside from motor speech. Importantly, these findings were obtained in a cohort whose diagnoses were established a priori without the use of the MLSE–NL, demonstrating that this screening tool accurately captures clinically meaningful language profiles. Together with its short administration time and error-based profile system, the results support the MLSE–NL as a possible useful tool for rapid triage of language impairment in Dutch-speaking patients with PPA. The MLSE–NL can be administered in a research or clinical setting without requiring detailed expertise in PPA diagnostics, although we advocate for familiarity with the basics of aphasia and training by experienced staff as was the case in this study. Furthermore, we also developed a normative dataset consisting of 276 controls for a range of age groups and education levels, which will facilitate clinical uptake. In controls, older age and male sex were associated with lower scores, and more years of education was associated with higher scores. Similar to observations regarding total MoCA score made in a Dutch control cohort [19], age, education and perhaps sex adjustment may prove useful in the interpretation of the MLSE–NL total score.

We believe that the MLSE–NL provides a useful tool to carry out an initial screening for language deficits by non-PPA experts, as well as use by clinicians with an expertise in diagnosing PPA (alongside a more comprehensive language battery). We propose that the MLSE act as an indicator that a patient should be flagged for further evaluation in a specialized center, rather than the end of the diagnostic pipeline. The MLSE–NL could potentially be useful for monitoring speech and language over time both in a clinical or a clinical trial setting, by assessing the progression of the different domains of speech and language. The large normative dataset provided in this manuscript offers a demographically matched comparison for clinicians to assess deviations from the norm within each domain of the MLSE–NL, unlike some other neuropsychological tests adapted to Dutch which were not validated as extensively in Dutch speakers.

The MLSE–NL showed comparable results to the original English version as well as to the adaptations in Spanish and Greek [7–9]. The language domain score profiles in the variants showed similar trends in all three languages, with the canonical variants presenting with the expected deficits, more motor speech errors in nfvPPA, more working memory errors in lvPPA, and more semantic knowledge errors in svPPA. The decision tree generated in the English dataset had an accuracy of 91%, while the Dutch had an accuracy of 83%. This difference may be due to differences in the Dutch language such as a richer morphological structure compared to English, or due to inclusion of patients who are either at a very early disease stage or further along in the disease and, therefore, display deficits in a wider range of speech and language domains. Likewise, the ROC AUC is similar in the three languages. The AUC in the English version when differentiating the different variants from controls, ranges from 0.89 to 0.99 (depending on the domain score and the PPA variant). The Spanish version of the MLSE has an AUC of 0.95 when comparing all PPA patients to controls based on MLSE total score, while the Dutch version has an AUC of 0.96–0.99 when comparing to controls depending on the PPA variant. The Dutch adaptation thus confirms the ability of the MLSE–NL to differentiate patients with PPA with high sensitivity and specificity in a new language. While the results show that classification into the canonical variants becomes less accurate with a more heterogeneous sample, the test still provides a precise description in linguistic terms of the nature of each patient’s deficit, which could be valuable in studying these syndromes longitudinally, as well as in helping patients and families understand and overcome their communication problems. The individual-level neurolinguistic profiles derived using MLSE–NL are neurobiologically valid, even though they do not completely align with the classification of PPA variants. Prior neuroimaging research has demonstrated that the relative proportion of error types in PPA is closely linked to specific cortical atrophy patterns [20] and regional hypometabolism [21]. For instance, in both studies the occurrence of semantic errors in PPA patients correlated with abnormalities in the left fusiform gyrus, the inferior and middle temporal gyri, across PPA variants.

The validation of the MLSE–NL alongside the existing language-specific version is important, because increasing evidence suggests that PPA manifests differently across languages [2, 4–6]. Investigations of PPA in English, Italian, and Chinese have found distinct symptom clusters depending on the language in question, with consistent semantic clusters but more heterogeneity in nfvPPA and lvPPA [22]. Studies in Italian- and English-speaking patients with nfvPPA have also shown language-specific differences with more syntactic errors in Italian-speaking patients compared to more distortions in English-speaking patients [4]. Cross-linguistic differences have also been reported in studies of Chinese-speaking patients, where language-specific features such as lexical tone and logographic writing systems produce distinct symptom profiles and error patterns compared with those observed in English-speaking cohorts [6, 23]. Studies in a number of different languages also show consistently lower scores in PPA patients in non-English languages compared to equally impaired patients (based on MRI atrophy patterns) in different nonlanguage tests [24]. This growing body of work underscores that diagnostic frameworks derived largely from English-speaking populations may not perform equally well across languages, and highlights the need for validated, language-specific assessment tools.

A strength of the MLSE–NL study is the inclusion of the PPA–NOS group. Many previous studies using the MLSE have not included this PPA variant before. Though a small group of patients (11/90 PPA patients, which is consistent with previously reported proportions of PPA–NOS), examination of the deficits in this group yields valuable insights. The definition of this group was kept broad, including all patients that did not fit within one of the canonical variants, rather than including only the “mixed” variant, as defined by Mesulam with both semantic deficits and agrammatism [25]. Accordingly, this group displayed deficits in all domains, aside from motor speech. The range of this group, however, was not dramatically larger than the other groups, with the nfvPPA remaining the most heterogeneous. The addition of this group into the classifiers reduced the overall accuracy, which was expected, given that this group has such widespread deficits. This PPA–NOS group is a good illustration that the MLSE–NL can be used to provide a quantitative marker of speech and language changes, although MLSE–NL is not directly able to identify this variant as such, and we acknowledge that the PPA–NOS group’s sample size is modest. However, we see a potential use for MLSE–NL in screening for PPA–NOS and perhaps it could also be a useful tool to track the progression of the disease course as more domains of speech and language become affected. Future studies will be needed to validate the proposed use of MLSE–NL as a means to monitor neurolinguistic profiles and disease severity longitudinally.

One limitation in this study, inherent to the nature of PPA, is the heterogeneity in the severity of the disease progression in the patients. Patients were not excluded or included based on the number of years since diagnosis which led to a widespread in the dataset, with MLSE–NL total scores in the patient group ranging from 30 to 96 and disease duration ranging from 4.5 to 85 months. In addition the underlying pathology of the patients was not assessed for all patients and there is a possibility that the misclassified nfvPPA patients had Alzheimer’s Disease pathology. Another consequence of the heterogeneity, particularly in severely affected patients, is the difficulty in error typing as the disease progresses, particularly in differentiating motor speech errors and phonological structure errors. Dialect differences also remained an important consideration in scoring. The selected tasks were chosen to be applicable in the standard Dutch as used in both the Netherlands as well as Belgium. The guidelines outlined in the scoring guide were followed as closely as possible to maintain standardization in scoring.

Further investigation of the MLSE in other patient groups may yield interesting results and uses in the future. Preliminary studies using the MLSE in English patients with Parkinson's plus disorders have found a reduction in MLSE total scores, with an average of 80.5 in supranuclear palsy and 74 in corticobasal syndrome [26], well below the calculated normal cutoff. Changes in speech and language have been widely reported in Parkinson ’s disease and Parkinson’s plus disorders [2, 27] for which the MLSE will be useful for detecting and tracking. Future use of the MLSE with a level of automation in assessment and scoring may also prove helpful for more wide scale screening for speech and language deficits in PPA and related neurodegenerative disorders. This approach would complement the growing body of work on automated speech analysis in FTD and PPA, where acoustic and linguistic features automatically extracted from speech have shown promise for disease detection, differential diagnosis, and monitoring of disease progression [28–30].

## Conclusion

In this paper we validate the MLSE–NL, a Dutch adaptation of the original English MLSE, as a tool to triage patients with PPA. We demonstrate the abilities of the MLSE–NL to detect different variants of PPA with high sensitivity and specificity, comparable to the original English version. When evaluating the MLSE–NL’s performance to classify between the three canonical variants, the accuracy level of 83% was slightly below what was found for the English version. In addition we provide a normative dataset across a range of ages and education levels, available for use by clinicians. The canonical variants show predictable patterns in their deficits in the different domains of the MLSE–NL, with patients with svPPA being the most affected in the semantic domain, patients with nfvPPA in the motor speech domain, and patients with lvPPA in the working memory domain. Patients with PPA–NOS showed deficits in all domains aside from motor speech. The results confirm that the MLSE–NL can be valuable to facilitate the diagnosis of Dutch-speaking patients with PPA.

## Supplementary Information

Below is the link to the electronic supplementary material.

Supplementary Material 1

## Data Availability

MLSE–NL materials are available upon reasonable request. The datasets generated and/or analyzed during the current study are not publicly available due to privacy and ethical restrictions concerning the sharing of individual-level patient data. The data supporting the findings of this study are available from the corresponding author upon reasonable request, subject to applicable ethical and data protection requirements.

## References

[1] Sajjadi SA, Patterson K, Arnold RJ, Watson PC, Nestor PJ (2012) Primary progressive aphasia. Neurology 78(21):1670–1677. 10.1212/WNL.0b013e3182574f79 PMC3359509 22573633

[2] Coppieters R et al (2024) A systematic review of the quantitative markers of speech and language of the frontotemporal degeneration spectrum and their potential for cross-linguistic implementation. Neurosci Biobehav Rev 167:105909. 10.1016/j.neubiorev.2024.105909 39393594

[3] García AM, de Leon J, Tee BL, Blasi DE, Gorno-Tempini ML (2023) Speech and language markers of neurodegeneration: a call for global equity. Brain. 10.1093/brain/awad253 PMC10690018 37497623

[4] Canu E et al (2020) Speech production differences in English and Italian speakers with nonfluent variant PPA. Neurology 94(10):e1062–e1072. 10.1212/WNL.0000000000008879 PMC7238919 31924679

[5] Tee BL et al (2022) Tonal and orthographic analysis in a Cantonese-speaking individual with nonfluent/agrammatic variant primary progressive aphasia. Neurocase 28(1):1–10. 10.1080/13554794.2021.1925302 PMC9345301 34404317

[6] Tee BL et al (2024) More than a piece of cake: noun classifier processing in primary progressive aphasia. Alzheimers Dement 20(4):2353–2363. 10.1002/alz.13701 PMC11032580 38284802

[7] Patel N et al (2022) A `Mini linguistic state examination’ to classify primary progressive aphasia. Brain Commun. 10.1093/braincomms/fcab299 PMC8914496 35282164

[8] Matias-Guiu JA et al (2021) Spanish version of the Mini-Linguistic State Examination for the diagnosis of primary progressive aphasia. J Alzheimers Dis 83(2):771–778. 10.3233/JAD-34366355

[9] Papadopoulou V et al (1998) Classifying and monitoring primary progressive aphasia in the Greek population: a ‘Mini Linguistic State Examination (MLSE)’ tool. Medicina (Mex) 61(11):1998. 10.3390/medicina61111998 PMC12654791 41303834

[10] Marshall CR et al (2018) Primary progressive aphasia: a clinical approach. J Neurol 265(6):1474–1490. 10.1007/s00415-018-8762-6 PMC5990560 29392464

[11] Jack CR et al (2018) NIA-AA Research Framework: toward a biological definition of Alzheimer’s disease. Alzheimers Dement 14(4):535–562. 10.1016/j.jalz.2018.02.018 PMC5958625 29653606

[12] International Test Commission (2017) “The ITC Guidelines for Translating and Adapting Tests (Second edition).” Accessed: Sep. 25, 2026. [Online]. Available: https://www.intestcom.org/

[13] Keuleers E, Stevens M, Mandera P, Brysbaert M (2015) Word knowledge in the crowd: measuring vocabulary size and word prevalence in a massive online experiment. Q J Exp Psychol. 10.1080/17470218.2015.1022560 25715025

[14] Brysbaert M, Mandera P, McCormick SF, Keuleers E (2019) Word prevalence norms for 62,000 English lemmas. Behav Res Methods 51(2):467–479 10.3758/s13428-018-1077-9 29967979

[15] Bruffaerts R et al (2022) Left frontal white matter links to rhythm processing relevant to speech production in apraxia of speech. Neurobiol Lang 3(4):515–537. 10.1162/nol_a_00075 PMC10158569 37215340

[16] Keuleers E, Brysbaert M (2010) Wuggy: a multilingual pseudoword generator. Behav Res Methods. 10.3758/BRM.42.3.627 20805584

[17] Miyagawa T et al (2020) Utility of the global CDR® plus NACC FTLD rating and development of scoring rules: data from the ARTFL/LEFFTDS Consortium. Alzheimers Dement 16(1):106–117. 10.1002/alz.12033 PMC7202045 31914218

[18] Koo TK, Li MY (2016) A guideline of selecting and reporting intraclass correlation coefficients for reliability research. J Chiropr Med 15(2):155–163. 10.1016/j.jcm.2016.02.012 PMC4913118 27330520

[19] Kessels RPC et al (2022) Regression-based normative data for the Montreal Cognitive Assessment (MoCA) and its Memory Index Score (MoCA-MIS) for individuals aged 18-91. J Clin Med 11(14):4059. 10.3390/jcm11144059 PMC9318507 35887823

[20] Bruffaerts R et al (2020) Multivariate analysis reveals anatomical correlates of naming errors in primary progressive aphasia. Neurobiol Aging 88:71–82. 10.1016/j.neurobiolaging.2019.12.016 31955981

[21] Catricalà E et al (2020) Neural correlates of naming errors across different neurodegenerative diseases: An FDG-PET study. Neurology 95(20):e2816–e2830. 10.1212/WNL.0000000000010967 33004608

[22] Santi G et al (2025) Cross‐linguistic characterization of speech and language profiles of English, Chinese, and Italian Primary Progressive Aphasia patients: a data‐driven approach. Alzheimers Dement. 10.1002/alz70857_106103

[23] Tee BL, Lorinda Kwan-Chen LY, Chen TF, Yan CT, Tsoh J, Lung-Tat Chan A, Wong A, Lo RY, Lu CL, Wang PN, Lee Y (2022) Dysgraphia phenotypes in native chinese speakers with primary progressive aphasia. Neurology. 98(22):e2245-57 10.1212/WNL.0000000000200350 PMC9162166 35410909

[24] Fernandez-Romero L et al (2025) Cognitive profiles in primary progressive aphasia variants: a cross-cultural Australian and Spanish investigation. J Neurol Sci 472:123446. 10.1016/j.jns.2025.123446 40068241

[25] Mesulam M, Wieneke C, Rogalski E, Cobia D, Thompson C, Weintraub S (2009) Quantitative template for subtyping primary progressive aphasia. Arch Neurol 66(12):1545–1551. 10.1001/archneurol.2009.288 PMC2796598 20008661

[26] Henderson SK et al (2024) Lexical markers of disordered speech in primary progressive aphasia and ‘Parkinson-plus’ disorders. Brain Commun. 10.1093/braincomms/fcae433 PMC11630745 39659971

[27] Panozzo Chiomento M, Vender M, Delfitto D (2026) Language competence in Parkinson’s disease: a systematic review of 20 years of research. J Speech Lang Hear Res 69(1):253–272. 10.1044/2025_JSLHR-25-00205 41348928

[28] Ballard KJ, Savage S, Leyton CE, Vogel AP, Hornberger M, Hodges JR (2014) Logopenic and nonfluent variants of primary progressive aphasia are differentiated by acoustic measures of speech production. PLoS ONE 9(2):89864. 10.1371/journal.pone.0089864 PMC3938536 24587083

[29] Garcia AM et al (2022) Automated detection of speech timing alterations in autopsy-confirmed nonfluent/agrammatic variant primary progressive aphasia. Neurology 99(5):500–511. 10.1212/WNL.0000000000200750 PMC9421598 35914945

[30] Robin J et al (2023) Development of a speech-based composite score for remotely quantifying language changes in frontotemporal dementia. Cogn Behav Neurol 36(4):237–248 10.1097/WNN.0000000000000356 PMC10683975 37878468

