# Supplementary material for: The Dutch Mini-Linguistic State Examination (MLSE-NL): findings in Primary Progressive Aphasia (PPA)
Source: J Neurol. 2026 Oct 23;273(11):684. doi: 10.1007/s00415-026-14203-3 (PMC13694381; doi:10.1007/s00415-026-14203-3)
Supplement: Supplementary file 1 [file 415_2026_14203_MOESM1_ESM.docx]

**Supplementary materials**

Table 1. Demographics of selected controls for Random Forest analysis.

|  | Selected controls (n = 25) |
| --- | --- |
| Sex (% male) | 48.0% (12/25) |
| Age (M ± SD) | 68.3 ± 2.8 |
| Education years (M ± SD) | 15.4 ± 0.5 |
| MoCA score /30 (M ± SD) | 27.3 ± 2.0 |
| BNT /60 (M ± SD) | 56.4 ± 3.2 |

Table 2. Normative data: cumulative frequencies for each MLSE-NL domain

| Total Score |  |  |  |
| --- | --- | --- | --- |
| Score | Frequency | Cum. Freq | Cum. % |
| 83 | 1 | 1 | 0,4 |
| 87 | 2 | 3 | 1,1 |
| 89 | 1 | 4 | 1,4 |
| 90 | 1 | 5 | 1,8 |
| 91 | 8 | 13 | 4,7 |
| 92 | 7 | 20 | 7,2 |
| 93 | 4 | 24 | 8,7 |
| 94 | 10 | 34 | 12,3 |
| 95 | 10 | 44 | 15,9 |
| 96 | 22 | 66 | 23,9 |
| 97 | 40 | 106 | 38,4 |
| 98 | 58 | 164 | 59,4 |
| 99 | 58 | 222 | 80,4 |
| 100 | 54 | 276 | 100 |
|  |  |  |  |
| Motor Speech (/30) |  |  |  |
| Score | Frequency | Cum. Freq | Cum. % |
| 28 | 1 | 1 | 0,4 |
| 29 | 40 | 41 | 14,9 |
| 30 | 235 | 276 | 100 |
|  |  |  |  |
| Phonological Structure (/30) |  |  |  |
| Score | Frequency | Cum. Freq | Cum. % |
| 23 | 2 | 2 | 0,7 |
| 24 | 1 | 3 | 1,1 |
| 25 | 3 | 6 | 2,2 |
| 26 | 5 | 11 | 4 |
| 27 | 18 | 29 | 10,5 |
| 28 | 36 | 65 | 23,6 |
| 29 | 83 | 148 | 53,6 |
| 30 | 128 | 276 | 100 |
|  |  |  |  |
| Semantic Knowledge (/20) |  |  |  |
| Score | Frequency | Cum. Freq | Cum. % |
| 7 | 1 | 1 | 0,4 |
| 13 | 2 | 3 | 1,1 |
| 14 | 1 | 4 | 1,4 |
| 15 | 1 | 5 | 1,8 |
| 17 | 3 | 8 | 2,9 |
| 18 | 17 | 25 | 9,1 |
| 19 | 66 | 91 | 33 |
| 20 | 185 | 276 | 100 |
|  |  |  |  |
| Syntax (/10) |  |  |  |
| Score | Frequency | Cum. Freq | Cum. % |
| 4 | 1 | 1 | 0,4 |
| 5 | 1 | 2 | 0,7 |
| 6 | 1 | 3 | 1,1 |
| 7 | 2 | 5 | 1,8 |
| 8 | 17 | 22 | 8 |
| 9 | 84 | 106 | 38,4 |
| 10 | 170 | 276 | 100 |
|  |  |  |  |
| Working Memory (/10) |  |  |  |
| Score | Frequency | Cum. Freq | Cum. % |
| 4 | 1 | 1 | 0,4 |
| 5 | 2 | 3 | 1,1 |
| 6 | 1 | 4 | 1,4 |
| 7 | 14 | 18 | 6,5 |
| 8 | 15 | 33 | 12 |
| 9 | 44 | 77 | 27,9 |
| 10 | 199 | 276 | 100 |

Table 3. Normative data: score corresponding to each percentile for the MLSE-NL

| Percentile | Total  Score (/100) | Motor  Speech (/30) | Phonological  Structure (/30) | Semantic  Knowledge (/20) | Syntax (/10) | Working  Memory (/10) |
| --- | --- | --- | --- | --- | --- | --- |
| 1th | 88,5 | 29 | 24,8 | 13,8 | 6,8 | 5,8 |
| 5th | 92 | 29 | 27 | 18 | 8 | 7 |
| 10th | 94 | 29 | 27 | 19 | 9 | 8 |
| 25th | 97 | 30 | 29 | 19 | 9 | 9 |
| 50th | 98 | 30 | 29 | 20 | 10 | 10 |
| 75th | 99 | 30 | 30 | 20 | 10 | 10 |
| 90th | 100 | 30 | 30 | 20 | 10 | 10 |
| 95th | 100 | 30 | 30 | 20 | 10 | 10 |
| 99th | 100 | 30 | 30 | 20 | 10 | 10 |

Table 4a. MLSE-NL performance by age group (controls). Values: Mean (SD), Range, 2-SD cutoff, 5th percentile.

| Domain | Statistic | ≤39 (n=44) | 40-49 (n= 21) | 50–59 (n=81) | 60–69 (n=63) | 70–79 (n=57) | 80+ (n=10) |
| --- | --- | --- | --- | --- | --- | --- | --- |
| Total Score | Mean (SD) | 98.3 (2.2) | 96.6 (3.7) | 97.8 (2.1) | 97.7 (2.7) | 96.4 (2.8) | 96.2 (2.5) |
| Total Score | Range | 91–100 | 83–100 | 90–100 | 87–100 | 89–100 | 92–100 |
| Total Score | 2 SD cutoff | 94.0 | 89.2 | 93.5 | 92.3 | 90.9 | 91.2 |
| Total Score | 5th pct | 94.1 | 91.0 | 94.0 | 92.2 | 91.0 | 92.4 |
| Motor Speech | Mean (SD) | 29.9 (0.3) | 29.9 (0.4) | 30.0 (0.2) | 29.8 (0.4) | 29.8 (0.4) | 29.5 (0.5) |
| Motor Speech | Range | 29–30 | 29–30 | 29–30 | 28–30 | 29–30 | 29–30 |
| Motor Speech | 2 SD cutoff | 29.3 | 29.1 | 29.5 | 28.9 | 28.9 | 28.4 |
| Motor Speech | 5th pct | 29.0 | 29.0 | 30.0 | 29.0 | 29.0 | 29.0 |
| Phonological Structure | Mean (SD) | 29.5 (0.8) | 28.9 (1.4) | 29.2 (1.0) | 29.0 (1.5) | 28.6 (1.4) | 29.0 (1.1) |
| Phonological Structure | Range | 27–30 | 25–30 | 26–30 | 23–30 | 24–30 | 27–30 |
| Phonological Structure | 2 SD cutoff | 27.8 | 26.1 | 27.2 | 26.0 | 25.8 | 26.9 |
| Phonological Structure | 5th pct | 28.0 | 26.0 | 27.0 | 26.1 | 26.8 | 27.4 |
| Semantic Knowledge | Mean (SD) | 19.1 (2.1) | 19.6 (0.6) | 19.5 (0.9) | 19.6 (1.0) | 19.4 (1.1) | 19.7 (0.7) |
| Semantic Knowledge | Range | 7–20 | 18–20 | 14–20 | 13–20 | 13–20 | 18–20 |
| Semantic Knowledge | 2 SD cutoff | 14.9 | 18.4 | 17.7 | 17.6 | 17.3 | 18.4 |
| Semantic Knowledge | 5th pct | 17.1 | 19.0 | 18.0 | 18.1 | 18.0 | 18.4 |
| Syntax | Mean (SD) | 9.9 (0.4) | 9.2 (1.1) | 9.4 (0.9) | 9.7 (0.6) | 9.2 (0.9) | 9.6 (0.5) |
| Syntax | Range | 8–10 | 6–10 | 4–10 | 8–10 | 5–10 | 9–10 |
| Syntax | 2 SD cutoff | 9.1 | 7.0 | 7.7 | 8.5 | 7.3 | 8.6 |
| Syntax | 5th pct | 9.0 | 7.0 | 8.0 | 9.0 | 8.0 | 9.0 |
| Working Memory | Mean (SD) | 9.4 (1.2) | 9.1 (1.7) | 9.7 (0.7) | 9.6 (0.7) | 9.4 (0.9) | 8.5 (1.0) |
| Working Memory | Range | 5–10 | 4–10 | 6–10 | 7–10 | 7–10 | 7–10 |
| Working Memory | 2 SD cutoff | 7.0 | 5.7 | 8.3 | 8.2 | 7.6 | 6.6 |
| Working Memory | 5th pct | 7.0 | 5.0 | 8.0 | 8.0 | 7.0 | 7.4 |

Appendix 4b. MLSE-NL performance by education group. Values: Mean (SD), Range, 2-SD cutoff, 5th percentile.

| Domain | Statistic | ≤11 years (n=22) | 12 years (n=21) | 13–15 years (n=105) | 16-17 years (n=60) | >17 years (n=59) |
| --- | --- | --- | --- | --- | --- | --- |
| Total Score | Mean (SD) | 95.3 (3.5) | 96.1 (3.8) | 97.7 (2.3) | 98.2 (2.0) | 97.9 (1.7) |
| Total Score | Range | 87–100 | 83–100 | 91–100 | 90–100 | 92–100 |
| Total Score | 2 SD cutoff | 88.3 | 88.5 | 93.1 | 94.1 | 94.5 |
| Total Score | 5th pct | 89.1 | 91.0 | 92.1 | 95.0 | 94.9 |
| Motor Speech | Mean (SD) | 29.7 (0.5) | 29.8 (0.5) | 29.9 (0.3) | 29.9 (0.3) | 29.8 (0.4) |
| Motor Speech | Range | 29–30 | 28–30 | 29–30 | 29–30 | 29–30 |
| Motor Speech | 2 SD cutoff | 28.8 | 28.8 | 29.2 | 29.4 | 29.0 |
| Motor Speech | 5th pct | 29.0 | 29.0 | 29.0 | 29.0 | 29.0 |
| Phonological Structure | Mean (SD) | 28.4 (1.7) | 28.8 (1.7) | 29.2 (1.1) | 29.3 (0.9) | 29.1 (1.0) |
| Phonological Structure | Range | 23–30 | 24–30 | 25–30 | 26–30 | 25–30 |
| Phonological Structure | 2 SD cutoff | 25.0 | 25.5 | 27.1 | 27.4 | 27.0 |
| Phonological Structure | 5th pct | 26.0 | 25.1 | 27.0 | 27.9 | 27.0 |
| Semantic Knowledge | Mean (SD) | 19.0 (1.5) | 19.4 (1.4) | 19.6 (0.7) | 19.5 (1.8) | 19.6 (0.8) |
| Semantic Knowledge | Range | 13–20 | 14–20 | 17–20 | 7–20 | 15–20 |
| Semantic Knowledge | 2 SD cutoff | 15.9 | 16.6 | 18.2 | 16.0 | 17.9 |
| Semantic Knowledge | 5th pct | 18.0 | 18.0 | 18.0 | 18.0 | 18.9 |
| Syntax | Mean (SD) | 9.0 (1.2) | 9.4 (1.0) | 9.5 (0.7) | 9.7 (0.9) | 9.7 (0.5) |
| Syntax | Range | 5–10 | 6–10 | 7–10 | 4–10 | 8–10 |
| Syntax | 2 SD cutoff | 6.5 | 7.5 | 8.1 | 7.9 | 8.6 |
| Syntax | 5th pct | 7.0 | 8.1 | 8.0 | 9.0 | 9.0 |
| Working Memory | Mean (SD) | 9.3 (1.1) | 8.9 (1.4) | 9.5 (1.0) | 9.6 (0.8) | 9.7 (0.9) |
| Working Memory | Range | 7–10 | 4–10 | 5–10 | 7–10 | 5–10 |
| Working Memory | 2 SD cutoff | 7.0 | 6.1 | 7.5 | 8.1 | 8.0 |
| Working Memory | 5th pct | 7.0 | 7.0 | 7.0 | 8.0 | 8.0 |

Appendix 4c. MLSE-NL performance by sex. Values: Mean (SD), Range, 2-SD cutoff, 5th percentile.

| Domain | Statistic | Female (n=164) | Male (n=112) |
| --- | --- | --- | --- |
| Total Score | Mean (SD) | 97.7 (2.7) | 97.0 (2.5) |
| Total Score | Range | 83–100 | 91–100 |
| Total Score | 2 SD cutoff | 92.3 | 92.0 |
| Total Score | 5th pct | 92.0 | 91.0 |
| Motor Speech | Mean (SD) | 29.8 (0.4) | 29.9 (0.3) |
| Motor Speech | Range | 28–30 | 29–30 |
| Motor Speech | 2 SD cutoff | 29.0 | 29.2 |
| Motor Speech | 5th pct | 29.0 | 29.0 |
| Phonological Structure | Mean (SD) | 29.2 (1.2) | 28.8 (1.3) |
| Phonological Structure | Range | 23–30 | 24–30 |
| Phonological Structure | 2 SD cutoff | 26.7 | 26.3 |
| Phonological Structure | 5th pct | 27.0 | 27.0 |
| Semantic Knowledge | Mean (SD) | 19.6 (0.6) | 19.2 (1.7) |
| Semantic Knowledge | Range | 17–20 | 7–20 |
| Semantic Knowledge | 2 SD cutoff | 18.4 | 15.7 |
| Semantic Knowledge | 5th pct | 18.0 | 17.0 |
| Syntax | Mean (SD) | 9.5 (0.9) | 9.5 (0.7) |
| Syntax | Range | 4–10 | 7–10 |
| Syntax | 2 SD cutoff | 7.8 | 8.1 |
| Syntax | 5th pct | 8.0 | 8.0 |
| Working Memory | Mean (SD) | 9.5 (0.9) | 9.4 (1.0) |
| Working Memory | Range | 4–10 | 5–10 |
| Working Memory | 2 SD cutoff | 7.7 | 7.4 |
| Working Memory | 5th pct | 8.0 | 7.0 |

Figure 1. Decision tree with PPA-NOS included.

*Abbreviations: svPPA = semantic variant Primary Progressive Aphasia, nfvPPA = non-fluent variant Primary Progressive Aphasia, lvPPA = logopenic variant Primary Progressive Aphasia, PPA-NOS = Primary Progressive Aphasia not otherwise specified*
